# Differentiation of Hair Sheep Breeds Based on the Physiological and Blood Biochemical Changes in Response to Different Stressors Using Multivariate Analysis Techniques

**DOI:** 10.3390/ani13162643

**Published:** 2023-08-16

**Authors:** Dereje Tadesse, Amlan Kumar Patra, Ryszard Puchala, Ali Hussein, Arthur Louis Goetsch

**Affiliations:** 1 American Institute for Goat Research, Langston University, Langston, OK 73050, USA; dereje.gulich@langston.edu (D.T.); puchala@langston.edu (R.P.); ali.hussein@enmu.edu (A.H.); arthur.goetsch@langston.edu (A.L.G.); 2 Department of Animal Sciences, Debre Berhan University, Debre Berhan P.O. Box 445, Ethiopia

**Keywords:** hair sheep, stressor, physiological response, multivariate analysis

## Abstract

**Simple Summary:**

Multivariate analyses were performed to differentiate Dorper, Katahdin, and St. Croix hair sheep breeds based on the changes in different physiological and blood variables due to various stresses, i.e., high heat load, restricted feed intake, and limited drinking water availability. Results demonstrated that Dorper and Katahdin were clustered together, and were separated from St. Croix in all stress conditions. This suggested that the stress responses of Dorper and Katahdin are similar, and divergent from that of the resilient St Croix breed. Under heat stress conditions, skin temperature, panting score, rectal temperature, respiration rate, and blood urea nitrogen and oxygen concentrations were the significant discriminating variables in clustering the sheep into groups. In terms of restricted feed intake, blood triglyceride and cholesterol concentrations were the most significant factors for the separation of St. Croix from Dorper and Katahdin, whereas blood hemoglobin, osmolality, protein, and albumin were the most important discriminating traits under the limited water availability condition.

**Abstract:**

Physiological and blood measurement changes due to high heat load, restricted feed intake, and limited drinking water availability in 135 animals of three hair sheep breeds (Dorper, Katahdin, and St. Croix) were subjected to multivariate analysis techniques. The objective of this analysis was to evaluate the ability of these variables to separate individual hair sheep into groups based on adaptation characteristics in response to three physiological stressors and identify variables with greater discriminatory power. There were 16, 8, and 13 physiological and blood variables obtained from high heat load, restricted feed consumption, and water intake studies, respectively, for multivariate analysis. Physiological variables such as respiration rate, rectal and skin temperature, and panting score were measured only in the heat stress study. The results of the cluster and canonical discriminant analyses showed the presence of wide divergence (*p <* 0.05) between St. Croix and other breeds in their responses to high heat loads and restricted-feed- and -water-intake conditions. Dorper and Katahdin were grouped (*p >* 0.05) together based on the changes in physiological variables, which were separated (*p <* 0.05) from those of St. Croix as a resilient group. The stepwise discriminant analysis indicated that skin temperature, panting score, rectal temperature, respiration rate, and blood urea nitrogen and oxygen concentrations were the significant (*p <* 0.05) discriminating variables in clustering individual sheep into groups based on their responses to the high-heat-stress condition. Under the limited feed intake condition, the significant (*p <* 0.05) traits responsible for the separation of St. Croix from Dorper and Katahdin were blood triglyceride and cholesterol concentrations, whereas blood hemoglobin, osmolality, protein, and albumin were most important discriminating variables under the limited water intake condition. In conclusion, the results of the present study suggest that the stress responses of Dorper and Katahdin are similar and different from that of St. Croix. This finding can be useful information for future decisions in developing climate-resilient sheep through selective breeding.

## 1. Introduction

Climate change causes different unprecedented changes in the biosphere, atmosphere, and hydrosphere, which consequently exert biotic and abiotic stresses on livestock. This could lead to a vulnerability of livestock production systems. A report points out that the effects of climate change in the USA could cause a high heat load and a depletion of forage and/or water resources [1], and these physiological stressors could adversely impact livestock productivity. The adoption of effective climate-adaptive actions would be required to moderate the vulnerability of livestock farming. This change in climate is expected to bring a shift from cattle production to sheep and goat production in the USA as seen in other parts of the world due the a greater tolerance of harsh environments by sheep and goats compared to cattle [2,3]. Having evolved in tropical environments, hair sheep are considered better-adapted to more stressful production conditions than are temperate wool breeds. Dorper, Katahdin, and St. Croix are the major hair sheep breeds found in the USA believed to have different adaptation capacities in harsh environments.

Livestock including sheep can have a series of behavioral, physiological, biochemical, hormonal, and molecular changes to cope with different environmental stressors including heat load and natural resource (such as feed and water availability and grazing land quality) restriction [4,5,6]. Differences in these changes between and among distinct genetic groups of animals exposed to various stress conditions have been considered indicators of adaptation or resilience to the stressors [7,8]. With the impending effects of climate change, the importance of adaptability in livestock production will increase [8]. Different livestock breeds exhibit a wide range of resilience and plasticity, which is useful for adaptation to the climate crisis [9]. Therefore, a genetic selection program designed to improve adaptation or resilience is a logical strategy. It is important, however, to understand the existing variation in adaptation or tolerance among different breeds and species before implementing a selection program.

Previous efforts to characterize breeds based on adaptive and other traits often have been based largely on univariate analyses of variance. Univariate statistical analyses treat each variable separately and do not explain how the breeds under investigations differ when all measured variables are considered jointly [10]. Sustainable livestock production in the context of climatic change requires considerations of different stress factors together to understand their physiological adaptation mechanisms rather than one variable separately. Multivariate analysis techniques have been very useful in support of characterization studies on breeds, with examples being the studies by Yakubu et al. [11] on African goats and Legaz et al. [12] on Assaf sheep. Therefore, in this paper, physiological and blood variables with these three hair sheep breeds were analyzed via multivariate analyses in order to group animals based on their adaptation capacity to high heat load and restricted feed and water intake conditions, and to determine which traits are most important in discriminating between any breed differences.

## 2. Materials and Methods

### 2.1. Animals, Housing and Diet

Three experiments targeting heat tolerance with a high heat load index (i.e., a heat stress study), the minimization of energy use with a limited nutritional plane (i.e., restricted feed intake study), and water conservation with restricted availability (i.e., restricted drinking water intake study) were conducted at the American Institute for Goat Research, Langston University (USA), after approval by the Langston University Animal Care and Use Committee. Each experiment consisted of four separate trials using four different sets of sheep pertaining to different years and seasons due to limited facilities to accommodate 128–135 sheep in a single trial. Animals were vaccinated with Covexin^®^ 8 (Schering-Plough Animal Health, Kenilworth, NH, USA) before starting the experiments. A 50% concentrate pelleted diet with assumed dietary metabolizable energy (ME) and crude protein concentrations of 9.62 MJ/kg and 160 g/kg dry matter (DM), respectively, was the main diet fed to the animals during the studies.

#### 2.1.1. High Heat Load

Detailed experimental procedures for this heat load study were described by Tadesse et al. [13,14]. Briefly, in total, 46 Dorper, 46 Katahdin, and 43 St. Croix female sheep from 47 commercial farms were used. They were obtained in the summer of 2015 from four regions of the USA with different climatic conditions, representing ‘ecotypes’. The regions were the Midwest (portions of Iowa, Minnesota, Wisconsin, and Illinois), Northwest (primarily Oregon with one farm in southern Washington and another near Seattle), Southeast (Florida and one farm in southern Georgia), and central Texas. Age at the start of the trials averaged 3.3 ± 0.18 years, ranging from 2.6 to 3.7 years. Four trials of this study were conducted in the fall of 2015, spring and fall of 2016, and spring of 2017.

The entire study period in each trial was 10 weeks, with the first week for adaptation to the facility and thermoneutral conditions and the last week for readjustment to thermoneutral conditions. Animals were situated in a thermo-controlled room individually in elevated pens with a plastic-coated expanded metal floor at most times. The room was equipped with multiple natural gas heaters, humidifiers and misters, and fans to maintain the targeted heat load index (HLI) conditions. There was a plastic barrel for feed and a bucket for water fitted to the front of 36 individual pens. The target HLI during the daytime (07:00 to 19:00 h) was 70, 85, 90, and 95 and that in nighttime was 70, 70, 77, and 81 in weeks 2 of periods 1, 2, 3, and 4, respectively. In higher HLI periods, the level of the HLI during the nighttime was approximately 85% of the HLI during the daytime [15]. The black globe temperature (BG; °C) and relative humidity (RH; %) were recorded every 15 min with a heat stress meter (Model SD-2010, REED Instruments, Wilmington, NC, USA) situated in the center of the room. In addition, five HOBO U12-011 data loggers (Onset Computer Corp., Bourne, MA, USA) placed at different locations were used to continuously monitor temperature and relative humidity. One digital thermo-anemometer (Model SD-4207, REED Instruments) was used to verify the presence of little to no wind. The heat load index (HLI) was calculated as proposed by Gaughan et al. [16] for temperatures above 25 °C: HLI = 8.62 + 0.38 × RH + 1.55 × BG − 0.5 × WS + e^(2.4 − WS)^, where WS = wind speed (m/s; assumed zero) and e = the base of the natural logarithm. Additionally, a temperature–humidity index (THI) was calculated using the formula of Amundson et al. [17]: (0.8 × temperature in °C) + (RH/100) × (temperature in °C − 14.4) + 46.4). The measured HLI and THI values during the daytime were 76.5 and 67.3, 86.5 and 77.8, 90.9 and 82.9, and 94.4 and 85.7 in periods 1, 2, 3, and 4, respectively. A pelleted diet was fed at 53.3 g DM/kg body weight^0.75^, which was approximately 120% of an assumed ME requirement. Water was made available ad libitum.

#### 2.1.2. Restricted Feed Intake

Detailed procedures were described by Tadesse et al. [18,19]. The ewes used in the heat stress study were mostly used for a restricted feed intake study. There were 46 Dorper, 47, Katahdin, and 41 St. Croix sheep in the four animal sets. Four trials were conducted in the winter of 2015/2016, winter of 2016/2017, spring/summer of 2017, and summer/fall of 2017. Age at the start of the trials averaged 3.8 ± 0.18 years, ranging from 1.2 to 11.7 years.

Animals were housed in five 6.1 m × 5.6 m pens in an enclosed building that had a 6.1 m × 1.35 m area with a concrete floor and a 6.1 m × 4.25 m unpaved floor area. The pens were aligned in a row adjacent to one another. Each pen was fitted with eight or nine Calan gate feeders (American Calan, Inc., Northwood, NH, USA). There were maintenance and restricted periods for this study. During the maintenance period of 4 weeks in length, feed to all animals was offered at 44.4 g DM/kg BW^0.75^, which was assumed adequate to meet the average ME requirement for maintenance. In the 6 weeks of period 2 or the restricted period, the DM offered was limited to 55% of the DM consumed during the maintenance period on an individual-animal basis. Animals had continuous access to drinking water. Temperature and RH in the facility were recorded continuously with U12-011Hobo Temperature/RH Data Logger (Onset Computer Corp., Bourne, MA, USA) situated in the center of the pens. The mean THI values during the day and night were 61.8 and 56.6, respectively, in the maintenance period, and 65.6 and 60.7, respectively, during the feed restriction period. This implies that animals were not exposed to heat stress during this experiment [20].

#### 2.1.3. Restricted Water Intake

Detailed experimental procedures were described by Hussein et al. [21,22]. Most of the sheep used in the two experiments mentioned above were used for the restricted water intake study as well. In this study, 44 Dorper, 42 Katahdin, and 42 St. Croix sheep with ages ranging from 2.7 to 3.9 years were used. The four separate trials were performed in the winter/spring (January to April) of 2016, summer (June–August) of 2016, winter/spring (January to April) of 2017, and summer (July to September) of 2017. The animals were housed in the same room that was used for the heat stress study mentioned above without the heat stress condition. The experiment was 9 weeks in length with the first 2 weeks serving as a baseline period (period 1) when water was offered as a free choice for ad libitum consumption to all animals. Average water intake during period 1 was used to determine the amounts offered thereafter. In weeks 3 and 4 (period 2) and weeks 5 to 9 (period 3), the amounts of water offered were 75% and 50% of that consumed in period 1 for all animals, respectively. Water was dispensed in buckets fitted to the front of each pen. A pelleted diet was fed up to approximately 160% of an assumed ME requirement for maintenance. If refusals were present, an amount of approximately 120% of the amount consumed was allocated. The mean THI values were 62.7, 56.0, and 77.0 during trial 1, 3, and 4, respectively.

### 2.2. Measurements

For all studies, blood samples were collected via jugular venipuncture using 10 mL tubes with and without sodium heparin every week on Friday at 13:00 h. However, rectal temperature, skin temperature, respiration rate, and panting score were recorded every Thursday at 07:00, 13:00, and 17:00 h only for the heat stress study. Rectal temperature was recorded using a digital thermometer (Vicks^®^, Model V900G, Hudson, NY, USA). Skin temperature was measured using a noncontact infrared thermometer (Fisher Scientific™ Traceable™, Waltham, MA, USA) at the area of the right shoulder shaved at the beginning of the experiment. Respiration rate was recorded by counting movements of the flank for 15 s and converting the values into a 1 min basis. The painting score was determined on a 5-point scale with 0 as no stress/no panting and 5 as extreme stress/elevated panting, as described by Tadesse et al. [14].

Immediately after blood sampling, hemoglobin concentrations and the oxygen saturation of hemoglobin in heparinized blood were determined with OSM 3 Hemoximeter™ (Radiometer, Westlake, OH, USA), and blood oxygen concentration was calculated as described by Eisemann and Nienaber [23] for heat stress and restricted water intake studies. Packed cell volume (PCV) was also determined with heparinized tubes (Clay Adams, Parsippany, NJ, USA). Plasma and serum were obtained via centrifugation for 15 min at 3000× *g* and frozen at −20 °C. For the restricted drinking water intake study, plasma was collected and immediately analyzed for osmolality (OSM) via freezing point depression using an OSMETTETM model 5004 osmometer (Precision System Inc.; Natick, MA, USA). For all studies, the thawed serum samples collected in each week of each period were analyzed for lactate, albumin, blood urea nitrogen, cholesterol, creatinine, glucose, total protein, triglycerides, and thyroxine with Vet Axcel^®^ Chemistry Analyzer (Alfa Wassermann Diagnostic Technologies, West Caldwell, NJ, USA) in accordance with the manufacturer’s instructions. Concentrations of cortisol, aldosterone (Enzo Life Sciences (Farmingdale, NY, USA), and heat shock protein 70 (Wuhan Fine Biotech Co., Ltd., Wuhan, China) were determined with ELISA kits.

### 2.3. Statistical Analyses

Physiological and blood data measured from animals during the low- (control) and high-stress periods were used for the multivariate analyses. Data were standardized due to the different measurement scales used for the various variables for the multivariate analyses. In order to group animals based on differences that occurred due to stress factors (high heat load, restricted feed intake, and limited water availability), measures of physiological and blood variables taken from each animal during the high-stress periods were subtracted from those during control periods. This was required to remove differences among breeds and animals due to their own physiological variations rather than the stress factors involved. Multivariate analysis was then applied to the changes (i.e., differences between high-stress and control periods) in physiological and blood variables. Analyses were performed separately for each study using statistical analysis software [24]. The means and standard deviations (SD) of the changes were computed for all traits measured. Different multivariate statistical analyses, i.e., discriminant analysis (PROC DISCRIM), cluster analysis (PROC CLUSTER), and canonical discriminant analysis (PROC CANDISC), were employed using selected traits. The discriminant procedure was used to determine the percentage of correct assignments of each animal to its breed group. Canonical discriminant analysis was performed to obtain canonical variates, canonical coefficients, and Mahalanobis distances between the breeds based on the selected traits. The cluster analysis procedure was used to construct a dendrogram tree using the average linkage method to group animals based on their similarity in responding to heat stress. Additionally, stepwise discriminant analysis (PROC STEPDISC) was performed to identify variables contributing most to the differentiation among the three breed groups. The relative importance of the traits in discriminating the three breeds of sheep was assessed using the level of significance.

## 3. Results

### 3.1. Changes in Physiological Variables

The changes in the physiological and blood measurements of hair sheep breeds subjected to different stressors are presented in Table 1. Generally, the changes in magnitude of all physiological and some blood measurements were smaller for St. Croix than for Dorper and Katahdin sheep when subjected to high-heat and restricted feed and water intake conditions. For most variables, within-breed variability as measured via the standard deviation was lower for St. Croix than for Dorper and Katahdin sheep.

### 3.2. Custer Analysis

**Figure 1:**
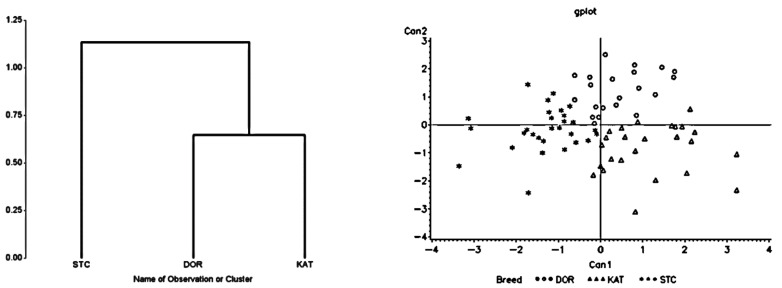
Dendrogram showing relationship (**left** panel) and canonical representation (**right** panel) of Dorper (DOR; circles), Katahdin (KAT; triangles), and St. Croix (STC; asterisks) sheep breeds subjected to high-heat-load condition.

**Figure 2:**
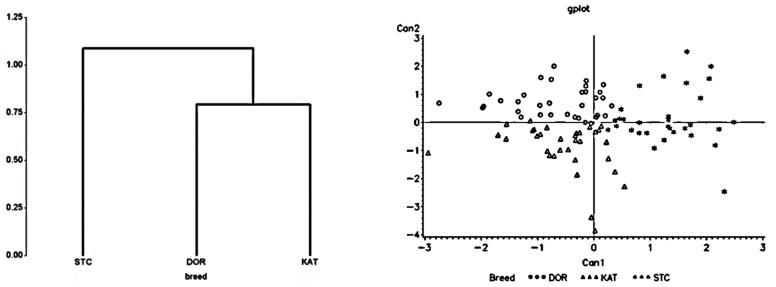
Dendrogram showing relationship (**left** panel) and canonical representation (**right** panel) of Dorper (DOR; circles), Katahdin (KAT; triangles), and St. Croix (STC; asterisks) sheep breeds subjected to restricted feed intake.

**Figure 3:**
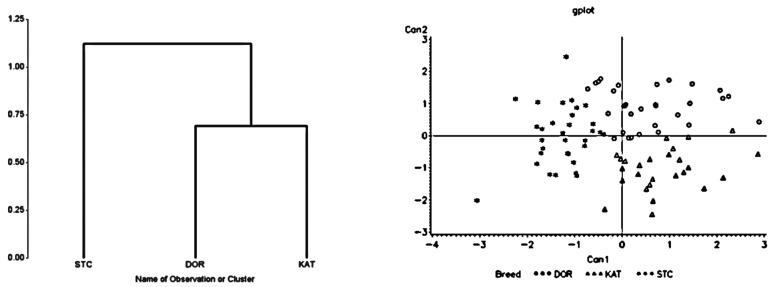
Dendrogram showing relationship (**left** panel) and canonical representation (**right** panel) of Dorper (DOR; circles), Katahdin (KAT; triangles), and St. Croix (STC; asterisks) sheep breeds subjected to limited water availability.

The results of the cluster dendrogram for high heat loads, restricted feed intake, and limited drinking water availability conditions are presented in Figure 1, Figure 2 and Figure 3, respectively. In all cases, two separate groups were formed by separating St. Croix from Dorper and Katahdin sheep, which were clustered as one group. There was a greater divergence between St. Croix and Katahdin sheep than between St. Croix and Dorper sheep. This was also confirmed with the results of the Mahalanobis distance analysis (Table 2). For each stress factor, there was a longer distance (*p <* 0.04) between St. Croix and Katahdin sheep followed by St. Croix and Dorper sheep, but the distance between Dorper and Katahdin sheep was short (*p >* 0.05). The longest distance between St. Croix and Katahdin (i.e., 5.23) sheep was seen when they were under high heat stress, whereas the shortest distance between Katahdin and Dorper sheep (i.e., 0.69) was seen under restricted feed intake conditions.

### 3.3. Canonical Discriminant Analysis

In the canonical discriminant analysis, two canonical variates (CAN 1 and CAN 2) were generated (Figure 1, Figure 2 and Figure 3). The first canonical variate (CAN 1), which accounted for 70.4, 73.4, and 73.6% of the total variances under a high heat load, restricted feed intake, and limited water availability conditions, respectively, was used to separate St. Croix from Dorper and Katahdin (*p* < 0.009) sheep. The second canonical variate (CAN 2), which accounted for between 26.4 and 29.6% of the total variance, did not contribute significantly (*p >* 0.05) in the discrimination process. The classification scores of individual sheep obtained from the discriminant analysis are depicted in Table 3. Overall, about 75.2, 55.8, and 66.9% of individual sheep were correctly assigned to their corresponding breed groups with classification errors of 24.8, 44.2, and 33.1% for heat stress, restricted feed consumption, and limited water availability, respectively. St. Croix had the highest classification scores for all stress factors, with the highest being for water restriction (i.e., 82.6%). On the other hand, Dorper had the lowest correct classification scores in all studies except heat stress studies with the lowest being for limited feed intake (i.e., 41.7%). The highest classification scores for Dorper (i.e., 75.0%) and Katahdin (i.e., 71.4%) sheep were estimated under the heat stress condition. There were high misclassification scores between Dorper and Katahdin in all stress factors with the highest being for the limited feed intake condition.

### 3.4. Stepwise Discriminant Analysis

The stepwise discriminant analysis showed that respiration rate, skin temperature, rectal temperature, panting score, and blood urea nitrogen and blood oxygen concentrations were identified as significant (*p <* 0.04) traits in discriminating the sheep breeds subjected to the heat load condition (Table 4). Conversely, only serum triglyceride and cholesterol concentrations had discriminatory power under limited feed intake (*p <* 0.04), whereas hemoglobin and protein concentrations and osmolality were the significant variables for differentiating between the breeds with limited water intake (*p <* 0.04). These variables had large loading scores on the first canonical functions (CAN 1) generated for each stress factor (Table 5). For the heat load condition, rectal temperature had the largest loading score, followed by respiration rate, and the lowest score was for skin temperature. For restricted feed intake, plasma triglyceride concentration had the highest loading score followed by cholesterol concentration, whereas the greatest loading score was for plasma albumin, followed by protein concentration, and the lowest score was for osmolality was generated in CAN 1 for restricted water intake.

## 4. Discussion

### 4.1. Descriptive Statistics and Breed Groups

Means and standard deviations for most traits, particularly for traits with high discriminating power, were high for Dorper and Katahdin sheep, implying that changes in responses to the stress factors and the variability of the responses were relatively high in these two breeds compared those in St. Croix sheep. In the previous report of Tadesse et al. [14], it was indicated that Dorper and Katahdin appeared to have higher variability than St. Croix did for some of physiological parameters, particularly for rectal and skin temperature. The variability of these variables within the Dorper and Katahdin sheep breeds indicates the importance of these variables in detecting differences within and between these breeds [25].

Multivariate analyses have been used in many studies to estimate divergence between breeds based on adaptation traits in response to stress factors using sheep and goats [11,26,27]. In the present study, physiological and blood variables related to adaptation to high heat loads, restricted feed intake, and limited water availability conditions were used to classify hair sheep breeds based on their responses to these stress factors and evaluate the contribution of the variables in the classification process. The results of all multivariate analyses in the present study indicate the existence of differences among the hair sheep breeds in their adaptation capacity in response to these three physiological stressors. Two groups of sheep were formed, one of St. Croix sheep (the more resilient group) and the other of Dorper and Katahdin sheep (the less resilient group), with St. Croix and Katahdin sheep being more divergent than St. Croix and Dorper sheep. This is slightly different from the previous reports of Tadesse et al. [14] and Hussein et al. [28] in which St. Croix sheep were closer to Katahdin than Dorper sheep based on a univariate analysis when they were under heat load and limited water intake conditions. The magnitude of divergence among the breeds was smaller under limited feed intake than with the other two stress factors. This could be due to the lower level of limited feed intake stress on animals compared to that of other stress factors. The responses of animals to various types of stress depend upon their intensities [29]. The previous report by Tadesse et al. [19] also shows that there were no significant differences between these breeds in blood constituent concentrations when subjected to limited feed intake.

The development of St. Croix sheep in a tropical environment with highly stressful conditions relative to the origins of Dorper and Katahdin sheep [30] may relate to the wider separation of St. Croix from Dorper and Katahdin sheep. Dorper is a South African breed developed from crosses of Dorset Horn and Blackhead Persian, whereas St. Croix was developed from West African hair sheep that were brought to the Caribbean [31,32]. St. Croix has been previously characterized as the most disease-resilient hair sheep breed in the United States and has shown exceptional parasite resistance when compared to Dorper, Suffolk or Katahdin sheep breeds [33,34]. Although Katahdin was originally developed from St. Croix during the second half of the 20th century [30], the results of the present study show that the distance between Katahdin and St. Croix sheep was higher than that between Katahdin and Dorper sheep. This might be attributed to some phenotypic or genetic commonalities between Katahdin and Dorper sheep breeds. For example, Dorper and Katahdin are different from St. Croix sheep in having a larger body size and relatively the same coat characteristics. As a result, Dorper and Katahdin could have similar physiological adaptation mechanisms in response to a stressful environmental condition.

### 4.2. High Heat Load

Large animals including Dorper and Katahdin sheep have difficulty in dissipating heat from the skin to the environment [35] and tend to minimize water loss through respiration because of their relatively smaller surface area to body mass ratio. According to Pacifici et al. [36], animals with a small body size have better adaptation responses to a hot climate. Relatedly, Dorper and Katahdin sheep, which are less dependent on sweating due to their thick hair and the presence of some wool under the coat, tend to increase their body temperature and respiration rate under heat stress. In the discriminant analysis, skin temperature, panting score, rectal temperature, respiration rate, and blood urea nitrogen and oxygen concentrations were found to be significant traits in differentiating between the more resilient (i.e., St. Croix) and less resilient groups (Dorper and Katahdin). It has been shown that respiratory rate and rectal temperature are good indicators of thermal stress and may be used to assess the adversity of the thermal environment [35,37,38]. In thermoneutral conditions, respiration accounts for about 20% of total body heat loss, which increases to about 60% at a higher temperature (35 °C) in sheep [39]. Rectal temperature, often used as a representative measurement of animal core temperature for practical purposes, is not easily affected by certain ranges of temperature in sheep as they are strict homeotherms [40]. The higher blood oxygen concentration in St. Croix sheep than that in Dorper and Katahdin sheep is suggestive of a deeper breathing pattern in the former as they were able to remove more carbon dioxide and saturate the blood more with oxygen [41]. The higher blood nitrogen level in St. Croix sheep under heat stress conditions makes them different from Dorper and Katahdin sheep because of their relatively higher DM intake as a percentage of body weight [14].

The stepwise discriminant analysis indicated that rectal temperature (the greater canonical coefficient in canonical variate 1) is the most important indicator for differentiating between hair sheep under heat stress, followed by respiration rate and panting score. Rectal temperature and respiration rate are the most important thermoregulatory traits in animals [35] and they are highly correlated, for example, r = 0.81 was reported by Li et al. [42], 0.75 was reported by Collier and Zimbelman [43], and 0.55 was reported by Martello et al. [44]. However, rectal temperature is perhaps more sensitive to high heat loads, which may then induce the other physiological sequela such as an increased respiration rate (followed by greater oxygen saturation) and panting score rate and reduced feed intake. Under conditions of a high level of heat load, when an animal body fails to maintain its heat balance via increased respiration and panting, body temperature measured via rectum (i.e., rectal temperature) increases and becomes important [26,35]. Physiological parameters such as rectal temperature and respiration rate in identifying heat-tolerant individual sheep or a heat-tolerant breed are vital because evaporative latent heat loss in sheep is less induced by sweating than by panting for dissipating heat from the body.

### 4.3. Restricted Feed Intake

Under restricted feed intake stress, the traits that most influenced the separation of St. Croix from Dorper and Katahdin sheep were blood triglyceride and cholesterol concentrations, reflecting that there were differences in energy metabolisms between the two groups when feed intake was restricted. Triglycerides are one of the most important sources of energy in animals, are affected by feed restriction [45,46] and can be used to predict energy status. A decrease in triglyceride concentration may occur due to feed restriction and explains the decline in substrate availability from the gastro-intestinal tract [47,48]. In the present study, the magnitude of decline in the triglyceride level in St. Croix sheep was lower probably because they were able to reduce their metabolic energy requirement and/or energy metabolism as described by Silanikove [20]. Relatedly, Atti et al. [49] noted that subcutaneous fat is the first energy depot to be mobilized when energy intake is deficient and hence the level of cholesterol in the blood increases because of lower feed intake [45,50]. The lower magnitude of an increase in cholesterol concentration for St. Croix vs. Dorper and Katahdin sheep is in accordance with the body condition score difference and the generally greater body fat levels for Dorper and Katahdin sheep [51].

Serum triglyceride concentration was the most significant indicator followed by cholesterol concentration for feed restriction. Under severe feed restriction, triglyceride concentration may increase to compensate for the maintenance energy deficit via the mobilization of fat reserves [45]. Body condition score and body fatness under feed restriction is reported to affect triglyceride concentration [52,53]. An increase in the level of serum triglycerides has been reported under conditions of feed restriction because of reduced lipoprotein lipase (which transfers triglyceride-derived fatty acids to adipose tissues for storage) activity and its mRNA expression in adipose tissues [54]. In the present study, the triglyceride concentration was a very important variable probably because of differences in energy metabolism and/or ME requirement for maintenance between St. Croix vs. Dorper and Katahdin sheep [18], with the former usually having a lower body condition score and body mass indices than the latter do [18,55,56]. A high body condition score (indicative of higher fat storage in the body) in Dorper and Katahdin sheep could cause greater fat mobilization from body reserves compared to a lower body condition score in St. Croix sheep in response to severe feed restriction [53,57,58].

### 4.4. Drinking Water Restriction

Differences in blood total protein and albumin concentrations are indicators of differences in dietary protein intake between the two groups. Serum total protein, and particularly albumin, are good indicators for predicting protein status in animals [59]. Albumin, in particular, serves as a labile protein reservoir providing a readily available source of amino acids until an alternative source is secured through diet or via mobilizing body sources such as skeletal muscle [60]. A decline in serum albumin concentration has been observed in ruminants with low dietary protein intake [59]. However, in line with the present study, many authors reported increases in blood albumin and globulin in sheep under water restriction conditions due to the lower plasma volume resulting from dehydration [61,62]. In the present study, the magnitude of increase in albumin and total protein were higher in Dorper than Katahdin and St. Croix sheep under the water restriction stress condition. Osmolality was another significant variable under limited water conditions. Water restriction lowers the plasma volume of blood that increases osmolality due to the higher levels of electrolytes in blood [63,64]. In the present study, osmolality was higher in Dorper and Katahdin but not in St. Croix sheep, indicating that the St. Croix breed was less affected by water restriction.

The stepwise discriminant analysis suggested that serum albumin, followed by protein concentration, was the most significant indicator and plasma osmolality was the least important indicator for restricted water intake among the significant variables. Albumin is the most significant discriminant variable under water restriction conditions because it plays an important role in osmoregulation and controlling fluid movement between different body parts as it is a major contributor to blood colloid osmotic pressure. According to Burton [63], the rates of albumin breakdown and synthesis are regulated in response to dehydration to maintain a normal intravascular colloid osmotic pressure and normal fluid distribution. Cortisol in body fluid is considered an important biochemical marker of stress, especially for acute conditions [65]. In the present study, it was not a determinant factor of any form of stress in the three hair sheep breeds, probably because stress was imposed for longer periods of time. It has been suggested that cortisol concentrations in body fluids and excreta may not reflect the overall stress response under chronic stress conditions [66].

## 5. Conclusions

Based on the multivariate analysis techniques used in the present study, the hair sheep breeds studied were distinctly clustered into two groups based on their responses to high-heat-load, and limited feed and water intake conditions. One group consisted of St. Croix sheep with better resilience and the other group consisted of Dorper and Katahdin with less resilience to the stress factors. Skin temperature, panting score, rectal temperature, respiration rate, and blood urea nitrogen and oxygen concentrations were significant discriminatory variables in classifying hair sheep breeds based on their adaptation capacity in response to high-heat-load conditions. On the other hand, under the limited feed intake condition, the traits that most influenced the separation of St. Croix from Dorper and Katahdin were blood triglyceride and cholesterol concentrations, while blood hemoglobin, osmolality, and protein and albumin concentrations were most important under the limited water intake condition. The multivariate analysis suggests that St. Croix sheep could be better adapted to adverse environmental conditions associated with climate change including high heat loads and the seasonal limited availability of drinking water and feed resources. The present results can be used for future decisions for developing climate-resilient sheep through selective breeding.

## Figures and Tables

**Table 1:** Changes in responses of hair sheep breeds to high heat load, and limited feed and water intake conditions.

Variable	Dorper		Katahdin		St. Croix	
	Mean	SD	Mean	SD	Mean	SD
High heat load						
Rectal temperature (°C)	0.47	0.36	0.44	0.25	0.27	0.27
Skin temperature (°C)	3.55	1.30	3.96	1.29	3.18	1.19
Respiration rate (breath/min)	92.0	28.6	86.3	31.1	82.4	24.3
Panting score (0–5 score)	1.05	0.34	1.08	0.39	0.87	0.23
Lactate (mg/dL)	−2.40	12.6	−1.55	10.9	0.45	7.03
Albumin (g/dL)	−0.057	0.29	−0.10	0.35	−0.015	0.28
Urea nitrogen (mg/dL)	−0.59	2.09	−1.41	3.74	0.73	3.18
Creatinine (mg/dL)	0.071	0.15	0.069	0.14	0.069	0.15
Glucose (mg/dL)	0.50	8.46	−1.41	8.64	−1.05	8.73
Total protein (g/dL)	−0.25	0.82	−0.42	0.97	−0.21	0.82
Triglyceride (mg/dL)	1.39	6.59	2.44	6.65	−0.39	5.24
Cholesterol (mg/dL)	−1.73	14.1	−2.12	14.2	−3.69	12.1
Cortisol (ng/mL)	−0.04	1.06	−0.05	1.19	0.09	0.065
Thyroxine (µg/dL)	0.13	0.94	−0.28	1.18	0.16	0.78
Heat shock protein (ng/mL)	0.03	0.88	0.04	1.05	−0.08	1.09
Packed cell volume (%)	−1.92	3.64	−2.08	3.49	−1.42	3.19
Hemoglobin concentration (g/dL)	−0.69	1.29	−0.98	1.38	−0.65	0.99
Oxygen saturation (%)	16.0	14.1	13.9	11.3	11.4	13.0
Oxygen concentration (mmol/L)	1.65	2.45	1.14	2.19	1.85	2.09
Restricted feed intake						
Lactate (mg/dL)	−5.13	6.98	−1.29	6.53	−3.67	7.15
Albumin (g/dL)	−0.002	0.19	0.005	0.22	0.006	0.16
Urea nitrogen (mg/dL)	−3.02	2.22	−2.83	1.96	−3.05	2.45
Creatinine (mg/dL)	0.13	0.13	0.14	0.16	0.11	0.13
Glucose (mg/dL)	0.14	7.34	1.62	11.3	−1.40	9.45
Total protein (g/dL)	0.13	0.52	0.008	0.49	−0.14	0.42
Triglyceride (mg/dL)	−6.08	5.98	−6.44	5.97	−3.66	5.09
Cholesterol (mg/dL)	13.9	15.5	14.5	18.1	8.13	9.37
Cortisol (ng/mL)	0.051	0.88	−0.17	1.13	0.14	0.96
Limited water intake						
Lactate (mg/dL)	−1.38	7.31	−3.37	7.55	−3.76	5.98
Albumin (g/dL)	0.15	0.29	0.04	0.39	0.12	0.24
Urea nitrogen (mg/dL)	1.52	4.19	1.05	6.09	1.11	3.09
Creatinine (mg/dL)	0.038	0.17	0.04	0.17	0.019	0.084
Glucose (mg/dL)	0.64	11.3	−1.12	9.81	2.42	8.56
Total protein (g/dL)	0.46	0.98	0.09	0.91	−0.044	0.91
Triglyceride (mg/dL)	7.81	12.3	4.49	8.29	4.00	7.72
Cortisol (ng/mL)	−0.05	1.29	0.13	0.81	−0.09	0.79
Aldosterone (pg/mL)	0.16	0.92	0.04	0.71	−0.24	1.31
Cholesterol (mg/dL)	13.5	16.4	9.05	15.9	12.7	11.9
Packed cell volume (%)	0.16	4.00	−0.65	3.35	2.39	1.99
Hemoglobin concentration (g/dL)	0.29	1.34	−0.06	1.05	0.79	0.88
Oxygen saturation (%)	2.42	12.9	2.25	11.3	6.62	10.4
Oxygen concentration (mmol/L)	0.49	2.68	0.24	2.48	1.52	2.45
Osmolality (mosmol/kg)	1.81	5.84	1.76	5.69	−0.90	4.55

SD, standard deviation.

**Table 2:** The Mahalanobis distances (above the diagonal) and their *p*-values (below the diagonal; also in bold font) estimated between the hair breeds.

	High Heat Load			Restricted Feed Intake			Limited Water Intake		
	DOR	KAT	STC	DOR	KAT	STC	DOR	KAT	STC
DOR		2.81	3.34		0.69	1.60		1.25	3.13
KAT	**0.202**		5.23	**0.301**		1.87	**0.422**		3.68
STC	**0.036**	**0.003**		**0.01**	**0.004**		**0.007**	**0.006**	

DOR, Dorper; KAT, Katahdin; STC, St. Croix.

**Table 3:** Percent (%) of individual animals classified into three breed groups based on their responses to different types of stress factors.

	Dorper	Katahdin	St. Croix	Error (%)
High heat load				
Dorper	75.0	12.5	12.5	25.0
Katahdin	14.3	71.4	14.3	28.6
St. Croix	10.3	10.3	79.3	20.7
Restricted feed intake				
Dorper	41.7	27.8	30.6	58.3
Katahdin	30.3	63.6	9.1	36.4
St. Croix	17.2	20.7	62.1	37.9
Limited water intake				
Dorper	56.6	20.0	19.4	43.4
Katahdin	26.9	61.5	11.5	38.5
St. Croix	17.4	0.00	82.6	17.4

**Table 4:** Physiological and blood biochemical traits with more discriminating power in differentiating between three hair breeds of sheep exposed to different types of stress factors.

Variables Selected	Partial *R*^2^	F Value	*p >* F *	Wilks’ Lambda	*p <* Lambda
High heat load					
Skin temperature	0.106	4.15	0.019	0.784	0.002
Panting score	0.093	3.52	0.035	0.711	0.001
Rectal temperature	0.106	4.04	0.022	0.635	<0.001
Respiration rate	0.111	4.18	0.019	0.565	<0.001
Blood urea nitrogen	0.124	5.02	0.009	0.876	0.009
Oxygen concentration	0.096	3.51	0.036	0.510	<0.001
Restricted feed intake					
Triglyceride	0.112	6.01	0.004	0.888	0.004
Cholesterol	0.067	3.35	0.039	0.829	0.001
Limited water intake					
Total hemoglobin	0.145	6.96	0.002	0.855	0.002
Osmolality	0.135	6.32	0.003	0.739	<0.001
Total protein	0.078	3.40	0.038	0.682	<0.001
Albumin	0.083	3.57	0.033	0.625	<0.001

* Only significant (*p* ≤ 0.05) variables are presented.

**Table 5:** Standardized canonical coefficients for the canonical discriminant function and the variance accounted for by each canonical variate.

Variables	Canonical 1	Canonical 2
High heat load		
Skin temperature	0.567	−0.271
Panting score	0.653	−0.253
Rectal temperature	0.839	0.599
Respiration rate	−0.689	0.513
Blood urea nitrogen	−0.649	0.172
Oxygen concentration	0.659	0.712
Variance accounted (%)	70.4	29.6
Restricted feed intake		
Cholesterol	−0.628	0.135
Triglyceride	1.049	0.171
Variance accounted (%)	73.4	26.6
Limited water intake		
Albumin	−0.962	−0.104
Total protein	0.877	0.549
Total hemoglobin	−0.431	3.046
Osmolality	0.401	−0.254
Variance accounted (%)	73.6	26.4

## Data Availability

Some data are presented in tables. Additional data can be made available from the corresponding upon reasonable request.
